# Sterols from Thai Marine Sponge *Petrosia* (*Strongylophora*) sp. and Their Cytotoxicity

**DOI:** 10.3390/md15030054

**Published:** 2017-02-23

**Authors:** Phanruethai Pailee, Chulabhorn Mahidol, Somsak Ruchirawat, Vilailak Prachyawarakorn

**Affiliations:** 1 Laboratory of Natural Products, Chulabhorn Research Institute, Kamphaeng Phet 6 Road, Laksi, Bangkok 10210, Thailand; pruethai@cri.or.th (P.P.); mahidol_natlab@cri.or.th (C.M.); somsak@cri.or.th (S.R.); 2 Chemical Biology Program, Chulabhorn Graduate Institute, Kamphaeng Phet 6 Road, Laksi, Bangkok 10210, Thailand; 3 Center of Excellence on Environmental Health and Toxicology (EHT), CHE, Ministry of Education, Bangkok 10400, Thailand

**Keywords:** *Petrosia* sp., sterol, cytotoxicity

## Abstract

Eight new sterols (**1**–**5** and **11**–**13**), together with eight known compounds (**6**–**10** and **14**–**16**) were isolated from marine sponge *Petrosia* sp. The structures of these compounds were elucidated on the basis of extensive spectroscopic analysis. The cytotoxicity of some compounds against a panel of human cancer cell lines is also reported.

## 1. Introduction

As part of our on-going search for biologically active metabolites from marine organisms [1,2,3], we have investigated a sponge species belonging to the genus *Petrosia* (*Strongylophora*) collected from the Similan Island, Thailand. Previously, various studies on the chemical constituents of *Petrosia* sp. led to the isolation of cyclosterols [4,5,6], polyacetylenic alcohols [7,8,9,10,11,12], meroditerpenes [13,14,15], 1,2-dihydroisoquinolines [16], halenaquinones [17], and pyridoacridine alkaloids [18]. Among them, some exhibited significant biological effects such as cytotoxicity [9,10,12,19,20,21], neurotrophic [11], antifouling [22] and antimicrobial activities [21], inhibitions against proteasome [15], protein Tyrosine phosphate 1B [13], cholinesterase [18], as well as inhibitory effects of the receptor activator of nuclear factor κB ligand (RANKL) induced osteoclastogenesis [17]. In this paper, we report the isolation and structure determination of eight new (**1**–**5** and **11**–**13**) and eight known (**6**–**10** and **14**–**16**) steroids from marine sponge *Petrosia* sp. and several of them were evaluated for their cytotoxicity against a panel of human cancer cell lines. The structures of eight new sterols have been established by extensive spectroscopic analysis, including 1D and 2D nuclear magnetic resonance (NMR) (distortionless enhancement by polarization transfer (DEPT), ^1^H-^1^H correlation spectroscopy (COSY), heteronuclear single quantum coherence (HSQC), heteronuclear multiple bond correlation (HMBC), and nuclear overhauser effect spectroscopy (NOESY)) spectroscopy.

## 2. Results

**Figure 1:**
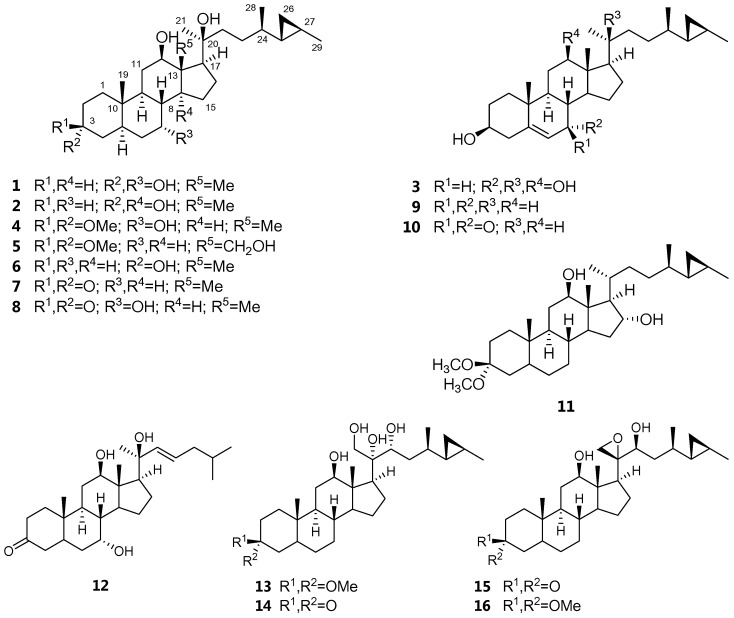
Chemical structures of compounds **1**–**16.**

*Petrosia* sp. (6.4 kg wet wt.) was collected by hand via scuba diving from the Similan Island in Thailand. The MeOH extract of the frozen sponge was partitioned between EtOAc and H_2_O, the EtOAc-soluble portion of the MeOH extract of *Petrosia* sp. exhibited significant cytotoxicity against various cancer cell lines (>67% inhibition of cell proliferation) at a concentration of 30 µg/mL. The EtOAc-soluble portion was then separated by sequential chromatographic techniques to afford eight new (**1**–**5** and **11**–**13**) and eight known (**6**–**10** and **14**–**16**) steroids (Figure 1).

**Figure 2:**
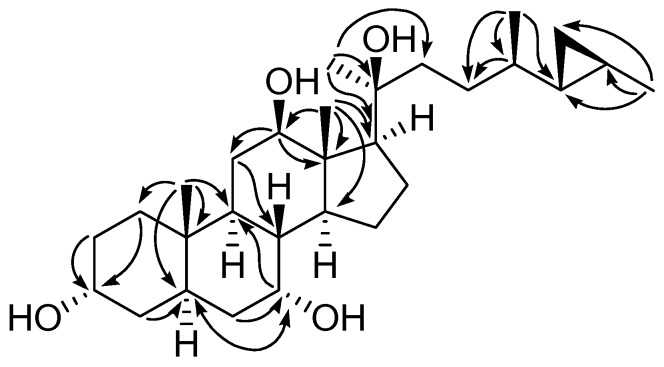
HMBC correlations of compound **1**.

**Figure 3:**
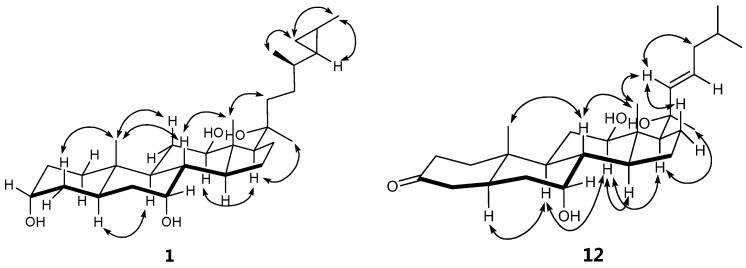
NOESY correlations of compounds **1** and **12**.

Compound **1** was obtained as a white amorphous powder, and the molecular formula was established as C_29_H_50_O_4_ by the atmospheric pressure chemical ionization-time of flight-mass spectrometer (APCI-TOF MS) at *m*/*z* 497.3414 [M + Cl]^−^ (calcd. for C_29_H_50_ClO_4_, 497.3403, see Figure S6). The infrared spectrophotometer (IR) spectrum exhibited a broad absorption band at 3280 cm^−1^, suggesting the presence of hydroxyl groups. The ^1^H NMR spectrum of **1** (Table 1, Figure S1) showed the upfield resonances of a cyclopropane ring at δ 0.20 (2H, m, H-25 and Hb-26), 0.10 (1H, m, Ha-26), and 0.54 (1H, m, H-27), three oxymethines at δ 4.27 (brs, H-3), 4.13 (brs, H-7), 3.76 (dd, *J* = 10.8, 2.6 Hz, H-12), three singlet methyls at δ 0.91 (CH_3_-19), 1.23 (CH_3_-18), and 1.35 (CH_3_-21), and two doublet methyls at δ 1.02 (*J* = 6.6 Hz, CH_3_-28 ) and 1.06 (*J* = 5.7 Hz, CH_3_-29). The ^13^C NMR (Table 2, Figure S2), DEPT, and HSQC spectra revealed the presence of twenty-nine carbons, comprising five methyls, ten methylenes, eleven methines, and three quaternary carbons. These data established the compound **1** as a C_29_-steroidal structure with a cyclopropane ring at C-25–C-27, and its NMR spectra revealed close similarity to those of aragusterol I (**6**), which was also isolated in this study and reported previously from the marine sponge *Xestospongia testudinaria* [22]. The only difference was the presence of signals for oxymethine (δ_H_ 4.13, s/δ_C_ 67.0) at C-7 in **1** instead of the signal for methylene in aragusterol I (**6**), suggesting that the C-7 position of **1** was substituted with the hydroxyl group. This assignment was supported by HMBC correlations (Figure 2 and Figure S4) from H-7 to C-5 (δ 45.6) and C-9 (δ 32.1) and H-6 to C-7 (δ 67.0). All of the ^1^H and ^13^C NMR signals of **1** (Table 1 and Table 2, Figures S1 and S2) were established by the HSQC, HMBC, ^1^H-^1^H COSY, and NOESY spectral analysis. The orientations of three hydroxyl groups at C-3, C-7, and C-12 were established by the ^1^H-^1^H coupling constants of H-3, H-7, and H-12, respectively. Two broad singlets of H-3 and H-7 were deduced as 3α-OH and 7α-OH configurations [21,22] while the dd (*J* = 10.8 and 2.6 Hz) of H-12 was assigned as 12β-OH-configuration [21]. In addition, α-orientation of hydroxyl group at C-3 was confirmed based on the similarity of ^13^C chemical shift of **1** (δ 65.8) with those reported for aragusterol I (δ 66.5, 3α-OH) [22]. The NOESY correlations (Figure 3 and Figure S5) among H-12 and H-17 and CH_3_-21 were assigned as β-orientations for both side chain at C-17 and hydroxyl group at C-20. The cyclopropane ring was assigned as possessing an *E*-geometry due to its NOESY correlation between H-25 and CH_3_-29. Moreover, the proton and carbon resonances of C-20–C-29 side chain of **1** were similar to those of aragusterols B (**7**) [23], I (**6**) [22], and xestokerol B (**8**) [21], indicating their identical relative configuration. In addition, the relative stereochemistry of the rings A–D (from C-1 to C-19) of **1** was deduced by the NOESY experiments as shown in Figure 3 and Figure S5. Therefore, compound **1** was identified as 26,27-cyclo-24,27-dimethylcholestan-3α,7α,12β,20β-tetraol (or 7α-hydroxyaragusterol I).

Compound **2** possessed the same molecular formula of C_29_H_50_O_4_ as that of **1**. Analysis of the ^1^H and ^13^C NMR spectral data of **1** and **2** (Table 1 and Table 2, Figures S1, S2, S7 and S8) revealed that a hydroxyl group signal present at C-7 in **1** was now present at C-14 in **2**, as the resonances at C-7 position were shifted from δ_H_ 4.13/δ_C_ 67.0 in **1** to δ_H_ 1.56 and 1.26 (m, each)/δ_C_ 26.5 in **2** and resonances at C-14 position from δ_H_ 1.93/δ_C_ 50.0 in **1** to δ_C_ 87.2 in **2**. The location of the hydroxyl group at C-14 in **2** was assigned based on the HMBC correlations from H-15 and CH_3_-18 to C-14 (δ 87.2). Concerning the configuration of 14-OH in **2**, the ^13^C chemical shifts of both C-9 and C-12 were significantly shifted upfield by ∆δC by approximately 6 ppm when compared with those reported for aragusterols I (**6**) [22] and B (**7**) [23] due to the γ-gauche effect [24]. These results established the hydroxyl group at C-14 as having an α-orientation. Consequently, compound **2** was identified as 26,27-cyclo-24,27-dimethylcholestan-3α,12β,14,20β-tetraol (or 14-hydroxyaragusterol I).

Compound **3** was isolated as a white amorphous powder. The ^1^H and ^13^C NMR spectra of **3** (Table 1 and Table 2, Figures S9 and S10) were closely similar to those of **1**, except for the orientation of the 3-OH group and the presence of signals for an additional double bond (δ_C_ 145.1 (C-5) and δ_H_ 5.91/δ_C_ 125.5 (C-6)) at C-5/C-6 in **3**. The HMBC correlations from H-4 and H-7 to C-5 (δ 145.1) and C-6 (δ 125.5) and CH_3_-19 to C-5 (δ 145.1), C-10 (δ 37.9), and C-9 (δ 42.6) indicated that the methylene group at C-6 in **1** was replaced by the double bond at C-5/C-6. The molecular formula of **3**, C_29_H_48_O_4_, had two mass units less than that of **1**, as determined from APCI-TOF MS at *m*/*z* 495.3252; [M + Cl]^−^ also supported this result. The orientation of 3-OH in **3** was determined by the carbon chemical shifts at C-3 and coupling constants of H-3. The downfield chemical shift of C-3 from δ_C_ 65.7 in **1** to 71.1 [22] in **3** and the multiplicity of H-3 at δ_H_ 3.77 as dddd (*J* = 10.6, 10.6, 5.6, 5.6 Hz) were assigned as 3β-OH orientation [4]. Thus, the new compound **3** was identified as 26,27-cyclo-24,27-dimethylcholest-5-ene-3β,7α,12β,20β-tetraol.

Compound **4** was obtained as an amorphous powder, and its molecular formula of C_31_H_54_O_5_ was established by the ESI-TOF MS at *m*/*z* 529.3858 [M + Na]^+^ (calcd for C_31_H_54_NaO_5_, 529.3864). After comparing the ^1^H and ^13^C NMR spectral data with that of compound **1** (Table 1 and Table 2), the proton and hydroxyl groups at C-3 in **1** were replaced by two methoxy groups in **4**, based on the ^1^H signals of two singlets at δ 3.16 and 3.07 and the ^13^C signal of ketal at C-3 (δ 100.7). Therefore, compound **4** was established as 26,27-cyclo-24,27-dimethylcholestan-3,3-dimethoxy-7α,12β,20β-triol (or 7α-hydroxyaragusterolketal I).

The molecular formula C_31_H_54_O_5_ of compound **5** was deduced from APCI-TOF MS. The ^1^H and ^13^C NMR spectral data closely resembled those of **4** except that the secondary hydroxyl group at C-7 and methyl group at C-13 in **4** were replaced by a methylene (δ_H_ 0.81 and 1.65) and a hydroxymethyl (δ_H_ 3.65 and 4.02/δ_C_ 60.8) group, respectively in **5**. The HMBC correlations from H-18 to C-12 (δ 80.6) and C-17 (δ 61.2) supported the position of the hydroxymethyl at C-13 of the sterol. Thus, compound **5** was elucidated as the new steroid 26,27-cyclo-24,27-dimethylcholestan-3,3-dimethoxy-12β,18,20β-triol.

The molecular formula of compound **11** was determined as C_31_H_54_O_4_ by APCI-TOF MS. The ^1^H and ^13^C NMR spectra of **11** (Table 2 and Table 3, Figures S15 and S16) were similar to those of compound **4**, suggesting a 12β-hydroxy group and dimethyl ketal at C-3. The main differences were that the position of the β-hydroxy group at C-7 in **4** was now at C-16 (δ_H_ 4.33/δ_C_ 74.2) and the hydroxy-substituted quaternary carbon at C-20 (δ_C_ 74.4) in **4** was replaced by the methine (δ_H_ 1.85/δ_C_ 32.3) group. The 16α-OH group in **11** was assigned on the basis of its correlations of H-16 with C-20 (δ 32.3) and C-14 (δ 51.3) in the HMBC correlations and the cross peak of H-17 and H-16 in the ^1^H-^1^H COSY together with the correlation between H-16 and CH_3_-18 in the NOESY spectrum. In addition, the relative stereochemistry of C-20–C-29 side chain at C-17 of compound **11** was confirmed by the similarity of the ^1^H and ^13^C chemical shifts to those of known petrosterol (**19**) [25] and 7-oxopetrosterol (**10**) [4]. The NOESY correlation between H-17 and H-12 further established the orientation of C-17 side chain as β-orientation. Thus, the structure of **11** was elucidated as 26,27-cyclo-24,27-dimethylcholestan-3,3-dimethoxy-12β,16α-diol.

Compound **12** was isolated as a white amorphous powder, and the molecular formula was established as C_27_H_44_O_4_ by APCI-TOF MS at *m*/*z* 467.2941 [M + Cl]^−^ (calcd for C_27_H_44_ClO_4_, 467.2934). The IR spectrum showed characteristic absorption bands of a hydroxyl group at 3518 cm^−1^ and a carbonyl group at 1713 cm^−1^. The ^1^H and ^13^C NMR spectra of the rings A–D of **12** (Table 2 and Table 3, Figures S17 and S18) were similar to those ofxestokerol B (**8**) [22], suggesting the presence of the carbonyl group at C-3 (δ 210.3), α-OH at C-7 (δ 66.3), and β-OH at C-12 (δ 78.0). The main difference was the signals due to the side chain (C-20–C-27) at C-17, which was assigned by the analysis of ^1^H-^1^H COSY and HMBC correlations. The ^1^H-^1^H COSY spectrum showed the cross peak between the olefinic H-23 (δ 5.94) and methylene H-24 (δ 1.99 and 2.03), which in turn coupled to a methine H-25 (δ 1.66) of the isopropyl group and showed the HMBC correlations from CH_3_-21 to C-17 (δ 64.1); 20-OH to C-20 (δ 73.9), C-21 (δ 31.6), and C-17 (δ 64.1); and both H-22 and H-23 to C-20 (δ 73.9). All of these suggested that the side chain of **12** was 1-hydroxy-1,5-dimethyl-2-hexenyl unit. The *trans* geometry of the double bond at C-22 and C-23 was established from the large coupling constants of 15.5 Hz. In addition, the orientation of the hydroxy group at C-20 could be established as β-OH from the cross peak between H-22 and CH_3_-18, H-17 and CH_3_-21 in NOESY analysis (Figure 3). Thus, compound **12** was identified as 7α,12β,20β-trihydroxycholesta-22*E*-en-3-one.

Compound **13** had the molecular formula C_31_H_54_O_6_ as determined by APCI-TOF MS. The NMR spectra of **13** (Table 2 and Table 3, Figures S19 and S20) were similar to those of a known xestokerol A (**14**), except for the replacement of the ketone at C-3 in **14** with the dimethyl ketal (δ_C_ 100.3). The HMBC correlations from 3-OMe, H-1, H-2, H-4, and H-5 to C-3 supported the location of the dimethyl ketal. Thus, compound **13** was identified as 26,27-cyclo-24,27-dimethylcholestan-3,3-dimethoxy-12β,21,20α,22α-tetraol (or 3-dimethyl ketal analogue of xestokerol A (**14**)) [21].

Besides these eight new compounds, the eight known structures, aragusterol I (**6**) [22], aragusterol B (**7**) [23], xestokerol B (**8**) [21], petrosterol (**9**) [25], 7-oxopetrosterol (**10**) [4], xestokerol A (**14**) [21], aragusterol A (**15**) [20], and aragusterolketal (**16**) [19] were isolated and identified by NMR techniques and comparison of their spectral data (^1^H and ^13^C NMR and [α]_D_) with literature values.

Although the 3-dimethyl ketal functionality of compounds **4** and **5** were assumed to have been artificially formed during the isolation and purification procedures, there have been some examples that the aragusteroketals A (**16**) [19] and B [24] possessing the 3-dimethyl ketal functionality have also been isolated from a marine sponge of *Xestospongia* sp. As an additional proof, the xestokerol B (**8**) and the aragusterol A (**15**) with carbonyl group at C-3 were subjected to conditions similar to those during the process of the isolation and purification for one month. No change in the thin layer chromatography (TLC) analyses was observed, suggesting that all isolated dimethyl ketal derivatives are naturally occurring.

In a previous study, sterols with a cyclopropane ring were reported to possess cytotoxicity toward various cancer cell lines [19,20,23]. In our study, several compounds (**1**–**4**, **6**–**8**, **10**, and **12**–**16**) were evaluated for their cytotoxicity using a panel of human cancer cell lines, including MOLT-3 (acute lymphoblastic leukemia), HepG2 (hepatocarcinoma), A549 (human lung cancer), HuCCA-1 (human chlolangiocarcinoma), HeLa (cervical carcinoma), and MDA-MB-231 (hormone-independent breast cancer) as well as a normal cell line, MRC-5 (normal human embryonic lung fibroblasts). As shown in Table 4, all of the tested compounds, except for sterol **15**, exhibited weak to moderate cytotoxicity, with the IC_50_ values in the range of 11.23–103.5 μM. The most potent, compound **15,** was cytotoxic, with the IC_50_ values of 7.10 and 6.11 μM against HepG-2 and HeLa cell lines, respectively, while exhibiting moderate cytotoxicity with the IC_50_ values of 12.84, 37.93, 37.58, and 18.01 μM against the other four cancer cell lines, MOLT-3, A549, HuCCA-1, and MDA-MB-231, respectively. It was noted that all of the tested compounds exhibited weaker cytotoxic activity than the positive control (etoposide or doxorubicin) and were noncytotoxic towards a normal cell line (MRC-5), with IC_50_ values greater than 37.68 μM.

## 3. Materials and Methods

### 3.1. General Experimental Procedures

UV–Vis spectra were obtained on a Shimadzu UV-1700 PharmaSpec Spectrophotometer (Shimadzu Corporation, Kyoto, Japan). Optical rotations were measured at the sodium D line (590 nm) on a JASCO 1020 digital polarimeter (Japan Spectroscopic Corporation, Tokyo, Japan). Fourier Transform infrared (FTIR) spectra were recorded with a universal attenuated total reflectance (UATR) attachment on a Perkin–Elmer Spectrum One spectrometer (PerkinElmer, Waltham, MA, USA). ^1^H-, and ^13^C- and 2D-NMR spectra were obtained at 600 and 150 MHz for ^1^H and ^13^C, respectively, on a Bruker AVANCE 600 spectrometer (Bruker Corporation, Billerica, MA, USA) with tetramethylsilane (TMS) (for CDCl_3_) and residual solvent peaks (for pyridine-*d*_5_) as internal standards. APCI-TOF MS were determined using a Bruker MicroTOFlc spectrometer (Bruker Corporation, Billerica, MA, USA). Column chromatography and preparative TLC were performed on normal-phase with Merck (Merck, Darmstadt, Germany) silica gel 60 (70–230 mesh ASTM) and PF_254_, respectively, and RP-18 reverse-phase silica gel (40–63 µM). Sephadex LH-20 (GE Healthcare, Uppsala, Sweden) was also used for column chromatography. TLC was carried out on silica gel 60 F_254_ plates (Merck, 0.2 mm). Medium pressure liquid chromatography (MPLC) was performed using a Büchi Pump Module C-605 and Büchi UV Monitor C-630 (Büchi, Flawil, Switzerland). High performance liquid chromatography (HPLC) was performed using a Thermoseparation products with spectra SYSTEM P4000 pump and coupled with PL-ELS 2100 evaporating light-scattering detector from Polymer Laboratories (settings: gas flow, 1.2 L/min; evaporation temperature, 90 °C; nebulizer temperature, 50 °C). For columns, Hichrom C18, 5 μm (21.2 × 250 mm) (Hichrom, Berkshire, UK), and Waters Symmetry C-18 prep (19 × 300 mm) stainless steel columns (Waters Corporation, Milford, MA, USA)were used.

### 3.2. Animal Material

The sponge, *Petrosia* sp., was collected from the Similan Islands in the Andaman Sea, Phang Nga, Thailand in February 2011, by hand via scuba diving. It was identified by Dr. Sumaitt Putchakarn, Institute of Marine Science, Burapha University, Bangsaen, Chonburi, Thailand. A voucher specimen (No. CRI 589) was deposited at the Laboratory of Natural Products, Chulabhorn Research Institute.

### 3.3. Extraction and Isolation

The sponge (wet weight ca. 6.4 kg) was extracted with MeOH and concentrated under reduced pressure. The MeOH extract was partitioned between EtOAc and water. The EtOAc-soluble portion (10 g) was chromatographed on silica gel column chromatography (CC), eluting with a gradient of increasing polarity (hexane, CH_2_Cl_2_, and MeOH) to afford thirteen fractions (A1–A13). Compound **9** (900 mg) was obtained from fraction A6. Fraction A8 (3.2 g) was further subjected to silica gel column eluted with hexane and acetone in a polarity gradient to provide twelve fractions (B1–B12). The combined fractions B4 (132 mg) and B5 (20 mg) were purified by preparative RP18-HPLC with MeOH/H_2_O (94:6, flow rate 12 mL/min) as eluent to give compound **10** (17 mg). Fraction B6 (1.0 g) was further chromatographed on Sephadex LH-20 CC (3 × 120 cm) by eluting with MeOH/CH_2_Cl_2_ (1:1) to yield five fractions (C1–C5). Fraction C3 (439.0 mg) was chromatographed on preparative RP-18 MPLC with solvents of MeOH/H_2_O (60% to 100% MeOH) over 240 min to give compounds **7** (74 mg) and **15** (34 mg). Compounds **11** (2.9 mg), **16** (15 mg), and **5** (2.9 mg) were obtained after purification of fraction B7 by preparative RP18-HPLC (eluent: CH_3_CN/H_2_O (90:10), flow rate 8.5 mL/min). Fraction A9 (2.5 g) was purified by gel permeation over a Sephadex LH-20 CC (3 × 120 cm), eluting with MeOH/CH_2_Cl_2_ (1:1) as mobile phase, to provide three fractions (D1–D3). A mixture of sterols (823 mg) was obtained from fraction D2 (2.2 g) after crystallization with CH_2_Cl_2_/MeOH. The mother liquor solution from fraction D2 was further purified by gel permeation over a Sephadex LH-20 CC (3 × 120 cm) with MeOH as eluent to yield six fractions (E1–E6). Fraction E4 (1.16 g) was chromatographed on preparative RP-18 MPLC with solvents of MeOH/H_2_O (70% to 100% MeOH) over 240 min to give twenty-two fractions (F1–F22). Fraction F11 was subjected to preparative RP18-HPLC with CH_3_CN/H_2_O (60:40, flow rate 12 mL/min) as eluent, to give compound **12** (18 mg). Fraction F19 (104.2 mg) was chromatographed on preparative RP18-HPLC (eluent: MeOH/H_2_O (85:15)) to provide compounds **8** (5 mg), **13** (10 mg), and **14** (6 mg). Compound **6** (17 mg) was obtained from fraction F22. Fraction A10 (716.3 g) was chromatographed on gel permeation over a Sephadex LH-20 CC (3 × 120 cm), eluting with MeOH as mobile phase, and then purified by chromatography on a silica gel column, eluting with a gradient of hexane and EtOAc (70%–100%) to provide five fractions (G1–G5). Fraction G4 (46.8 mg) was purified by preparative RP18-HPLC eluting with MeOH/H_2_O (85:15, flow rate 12 mL/min) as mobile phase to provide compound **2** (5 mg). Fraction G5 (139.3 mg) was chromatographed on preparative RP-18 MPLC with solvents of MeOH/H_2_O (65% to 100% MeOH) over 360 min to give twelve fractions (H1–H12). Compounds **3** (11 mg) and **1** (8 mg) were obtained after purification of fraction H6 and H10, respectively, by preparative RP-18 HPLC with MeOH/H_2_O (80:20 for compound **3** and 85:15 for compound **4**, flow rate 12 mL/min for both **3** and **4**) as eluent.

Compound **1**. White amorphous powder; [α]${}_{D}^{25}$ −15.30 (*c* 0.63, MeOH); IR (ATR) ν_max_: 3280, 2925, 2856, 2310, 1951, 1722, 1666, 1452, 1372, 1145, 1005 cm^−1^; ^1^H and ^13^C NMR data (see Table 1 and Table 2); APCI-TOF MS *m*/*z*: 497.3414 [M + Cl]^−^ (calcd. for C_29_H_50_ClO_4_, 497.3403).

Compound **2**. White amorphous powder; [α]${}_{D}^{29}$ −6.87 (*c* 0.53, CHCl_3_); IR (ATR) ν_max_: 3304, 2927, 2926, 2857, 1449, 1372, 1004 cm^−1^; ^1^H and ^13^C NMR data (see Table 1 and Table 2); APCI-TOF MS *m*/*z*: 497.3384 [M + Cl]^−^ (calcd. for C_29_H_50_ClO_4_, 497.3403).

Compound **3**. White amorphous powder; [α]${}_{D}^{29}$ −70.06 (*c* 0.53, MeOH); IR (ATR) ν_max_: 3239, 2926, 2855, 1734, 1455, 1374, 1056, 1015 cm^−1^; ^1^H and ^13^C NMR data (see Table 1 and Table 2); APCI-TOF MS *m*/*z*: 495.3252 [M + Cl]^−^ (calcd. for C_29_H_48_ClO_4_, 495.3247).

Compound **4**. White amorphous powder; [α]${}_{D}^{28}$ −18.28 (*c* 0.90, CHCl_3_); IR (ATR) ν_max_: 3230, 2951, 2918, 2870, 1720, 1452, 1373, 1362, 1243, 1175, 1141, 1116, 1044, 1018, 921, 750 cm^−1^; ^1^H and ^13^C NMR data (see Table 1 and Table 2); ESI-TOF MS *m*/*z*: 529.3858 [M + Na]^+^ (calcd. for C_31_H_54_NaO_5_, 529.3864).

Compound **5**. White amorphous powder; [α]${}_{D}^{26}$ −11.97 (*c* 0.30, CH_2_Cl_2_); ^1^H and ^13^C NMR data (see Table 2 and Table 3); APCI-TOF MS *m*/*z*: 507.4044 [M + H]^+^ (calcd. for C_31_H_55_O_5_, 507.4036).

Compound **11**. White amorphous powder; ^1^H and ^13^C NMR data (see Table 2 and Table 3); APCI-TOF MS *m*/*z*: 525.3708 [M + Cl]^−^ (calcd. for C_31_H_54_ClO_4_, 525.3716).

Compound **12**. White amorphous powder; [α]${}_{D}^{26}$ +33.70 (*c* 1.82, MeOH); IR (ATR) ν_max_: 3518, 3248, 2955, 2871, 1713, 1450, 1384, 1226, 1156, 1063, 1041, 1019, 974, 748 cm^−1^; ^1^H and ^13^C NMR data (see Table 2 and Table 3); APCI-TOF MS *m*/*z*: 467.2941 [M + Cl]^−^ (calcd. for C_27_H_44_ClO_4_, 467.2934).

Compound **13**. White amorphous powder; [α]${}_{D}^{26}$ +20.43 (*c* 0.80, CHCl_3_); IR (ATR) ν_max_: 3274, 2946, 2871, 1716, 1456, 1360, 1178, 1055, 970 cm^−1^; ^1^H and ^13^C NMR data (see Table 2 and Table 3); APCI-TOF MS *m*/*z*: 557.3624 [M + Cl]^−^ (calcd. for C_31_H_54_ClO_6_, 557.3614).

### 3.4. Cytotoxicity Assays

The cytotoxic activity toward a panel of mammalian cancer cell lines (HepG2, A549, HuCCA-1, HeLa, MDA-MB-231) were tested using the 3-(4,5-dimethylthiazol-2-yl)-2,5-diphenyltetrazolium bromide (MTT) assay [26], while the activity toward MOLT-3 cancer cell line was performed by the 2,3-bis-(2-methoxy-4-nitro-5-sulphenyl)-(2H)-tetrazolium-5-carboxanilide (XTT) assay [27]. Etoposide and doxorubicin were used as positive controls (Table 4).

## 4. Conclusions

The chemical investigation of Thai marine sponge *Petrosia* sp. led to the isolation of eight new (**1**–**5** and **11**–**13**) and eight known (**6**–**10** and **14**–**16**) sterols. Their structures were established by the basis of spectroscopic method. Some compounds (**1**–**4**, **6**–**8**, **10**, and **12**–**16**) were evaluated for their cytotoxicity using a panel of human cancer cell lines. The most potent, compound **15**, was cytotoxic, with the IC_50_ values of 7.10 and 6.11 μM against HepG-2 and HeLa cell lines, respectively.

## Figures and Tables

**Table 1:** ^1^H NMR (600 MHz) data of compounds **1**–**4** from marine sponge *Petrosia* sp.

Position	δ_H,_ mult. (*J* in Hz)			
	1 ^a^	2 ^b^	3 ^a^	4 ^b^
1	1.50, m	1.39, m	1.06, m	1.23, ddd (13.6, 13.6, 3.6)
		1.53, m	1.80, m	1.75, m
2	1.28, m	1.29, m	1.81 ^c^, m	1.46, m
	1.82, m	1.64, m	1.90, m	1.89, m
3	4.27, s	4.05, s	3.76, dddd (10.6, 10.6, 5.6, 5.6)	
4	1.61, m	1.40 ^c^, m	2.63, t (12.1)	1.37, m
		1.50, m	2.69, dd (13.0, 4.4)	1.69, m
5	1.92, m	1.56, m		2.30, t (13.3)
6	1.65, m	1.40 ^c^, m	5.91, d (4.9)	1.52 ^c^, m
		1.64, m		1.62, m
7	4.13, s	1.26, m	4.15, s	4.06, s
		1.56, m		
8	1.59, m	1.79, m	1.61, m	1.51 ^c^, m
9	2.92, m	1.40 ^c^, m	1.85, m	1.75, m
11	1.63, m	1.27, m	1.80 ^c^, m	1.57, m
	2.10, m	1.77, m	2.07, m	2.00, m
12	3.76, dd (10.8, 2.6)	3.97, dd (11.0, 4.9)	3.84, dd (10.9, 4.1)	3.72, dd (10.9, 4.1)
14	1.93, m		2.05 ^d^, m	1.85, m
15	1.43, m	1.47, m	1.48, m	1.40, m
	2.12, m	1.62, m	2.25, m	2.10, m
16	1.67, m	1.53, m	1.70, m	1.63, m
	1.78, m	1.82, m	1.82 ^c^, m	1.76, m
17	1.99, m	2.62, t (9.9)	2.05 ^d^, m	1.95, m
18	1.23, s	0.92, s	1.22, s	1.17, s
19	0.91, s	0.82, s	1.07, s	0.83, s
20				
21	1.35, s		1.37, s	1.32, s
22	1.78, m	1.60, m	1.80 ^c^, m	1.52, m
	2.12, m	1.81, m	2.15, t (12.9)	2.11, m
23	1.78, m	1.29, m	1.49, m	1.48, m
	1.97, m	1.54, m	2.00, m	1.97, m
24	0.70, m	0.65, m	0.72, m	0.69, m
25	0.20 ^c^, m	0.16 ^d^, m	0.21 ^e^, m	0.20 ^d^, m
26	0.10, m	0.10, m	0.11, m	0.10, m
	0.20 ^c^, m	0.16 ^d^, m	0.21 ^e^, m	0.20 ^d^, m
27	0.54, m	0.49, m	0.54, m	0.53, m
28	1.02, d (6.6)	0.92, d (6.0)	1.03, d (6.2)	1.01, d (6.7)
29	1.06, d (5.7)	1.03, d (6.0)		1.06, d (6.0)
3-OMe				3.07, s
3-OMe				3.16, s

^a^ Measured in pyridine-*d*_5_; ^b^ Measured in CDCl_3_; ^c–e^ overlapped with other signals.

**Table 2:** ^13^C NMR (150 MHz) data of compounds **1**–**5** and **11**–**13** from marine sponge *Petrosia* sp.

Position	δ_C_							
	1 ^a^	2 ^b^	3 ^a^	4 ^b^	5 ^a^	11 ^b^	12 ^a^	13 ^b^
1	32.9	32.3	37.6	35.4	34.9	35.4	38.7	35.0
2	29.9	29.0	32.4	28.9	28.25	28.3	38.4	28.3 ^c^
3	65.8	66.4	71.1	100.7	100.3	100.3	210.3	100.3
4	36.75	35.8	43.3	35.8	35.3	34.9	44.66	35.4
5	45.6	38.9	145.1	35.23	42.3	42.4	39.7	42.4
6	38.0	28.33	125.5	37.6	28.34	28.4	38.0	28.4 ^c^
7	67.0	26.5	64.6	66.7	31.6	31.5	66.3	31.4
8	39.1	36.8	37.1	39.0	34.6	33.7	38.8	33.9
9	32.1	46.4	42.6	45.2	53.1	52.8	44.71	52.7
10	36.79	36.3	37.9	36.3	35.7	35.7	36.1	35.7
11	29.7	28.27	30.0	30.0	31.2	31.1	30.3	29.1
12	78.4	71.8	78.1	78.2	80.6	79.8	78.0	78.0
13	49.1	52.7	48.8	49.0	50.5	49.3	49.1	49.3
14	50.0	87.2	49.1	49.8	54.9	51.3	49.5	54.5
15	23.5	33.1	24.0	23.5	23.4	36.1 ^c^	23.6	22.8
16	25.6	24.0	25.8	25.6	23.9	74.2	25.1	23.5
17	66.0	57.6	65.8	65.9	61.2	67.9	64.1	54.2
18	10.2	12.4	10.2	10.2	60.8	9.1	10.0	9.7
19	10.6	10.9	18.5	10.9	11.58	11.49	10.4	11.5
20	74.4	75.5	74.5	74.4	74.6	32.3	73.9	76.3
21	28.6	28.9	28.6	28.6	29.0	22.5	31.6	66.4
22	35.2	34.4	35.2	35.15	37.0	32.7	137.6	75.1
23	32.5	31.9	32.5	32.5	32.0	36.2 ^c^	125.8	37.7
24	39.5	39.0	39.5	39.5	38.8	38.7	42.4	34.7
25	27.7	27.1	27.7	27.7	27.1	27.3	28.9	27.9
26	11.9	11.5	11.9	11.9	11.64	11.53	22.5 ^c^	12.5
27	13.2	12.8	13.2	13.2	12.7	12.7	22.8 ^c^	12.3
28	20.6	20.1	20.6	20.7	19.8	19.9		18.7
29	19.3	19.1		19.3	19.1	19.1		19.1
3-OMe				47.3	47.45	47.4		47.38 ^d^
3-OMe				47.4	47.51	47.5		47.43 ^d^

^a^ Measured in pyridine-*d*_5_; ^b^ Measured in CDCl_3_; ^c,d^ interchangeable.

**Table 3:** ^1^H NMR (600 MHz) data of compounds **5** and **11**–**13** from marine sponge *Petrosia* sp.

Position	δ_H,_ mult. (*J* in Hz)			
	5 ^a^	11 ^b^	12 ^a^	13 ^b^
1	1.11, ddd (13.6, 13.6, 3.7)	1.30, m	1.30, ddd (13.4, 13.4, 4.8)	1.05, ddd (13.6, 13.6, 3.7)
	1.62, m	1.55, m	1.88, m	1.60, m
2	1.48, m	1.40, m	2.28, m	1.43, ddd (14.0, 14.0, 4.0)
	1.89, ddd (12.9, 12.9, 3.8)	1.89, dq (14.1, 3.2)	2.36, m	1.88, br dd (2.9, 14.0)
4	1.30, m	1.12, m	2.12, m	1.28, m
			2.32, m	
5	1.31, m	1.30, m	2.43, m	1.29, m
6	1.28, m	1.20, m	1.56, ddd (13.7, 13.7, 2.0)	1.25, m
		1.28, m	1.65, m	
7	0.81, m	0.88, m	4.07, brs	0.88, m
	1.65, m	1.64, m		1.67, m
8	1.32, m	1.31, m	1.51, ddd (11.2, 11.2, 2.2)	1.30, m
9	0.85, m	0.88, m	1.76, m	0.84, m
11	1.57, m	1.25, m	1.65, m	1.26, m
	1.93, ddd (13.6, 4.0, 4.0)	1.67, m	2.04, m	1.73, m
12	3.50, dd (11.0, 4.0)	3.53, dd (10.9, 4.5)	3.75, dd (10.9, 4.3)	3.38, dd (11.0, 4.4)
14	1.02, m	1.30, m	1.87, m	1.76, m
15	1.01, m	1.42, m	1.40, m	1.50, m
	1.66, m	1.67, m	2.10, m	1.75, m
16	1.75, m	4.33, t (7.4)	1.76, m	1.20, m
				1.69, m
17	1.75, m	1.40, m	2.03, m	0.96, m
18	3.65, d (12.0)	0.71, s	1.07, s	0.80, s
	4.02, d (12.0)			
19	0.84, s	0.79, s	1.00, s	0.80, s
20		1.85, m		
21	1.44, s	1.09, d (6.9)	1.45, s	3.83, d (11.5)
				3.98, d (11.5)
22	1.61, m	0.88, m	6.00, d (15.5)	3.82, d (10.6)
	1.90, m	1.26, m		
23	1.28, m	1.44, m	5.94, ddd (15.0, 7.5, 7.5)	1.35, m
	1.47, m	1.66, m		1.67, m
24	0.70, m	0.65, m	1.99, m	0.93, m
			2.03, m	
25	0.19, m	0.16 ^c^, m	1.66, m	0.25, m
26	0.11, m	0.13 ^c^, m	0.92, d (6.6)	0.16, m
	0.18, m			0.25, m
27	0.51, m	0.46, m	0.91, d (6.6)	0.53, m
28	0.93, d (6.7)	0.91, d (6.7)		0.95, d (6.0)
29	1.02, d (6.0)	1.00, d (6.0)		1.03, d (6.0)
3-OMe	3.14, s	3.14, s		3.14, s
3-OMe	3.19, s	3.19, s		3.19, s

^a^ Measured in pyridine-*d*_5_; ^b^ Measured in CDCl_3_; ^c^ overlapped with other signals.

**Table 4:** Cytotoxicity data of pure compounds from the marine sponge *Petrosia* sp.

Compounds	Cell Lines (IC_50_, µM); Values Are Expressed as Mean ± S.D. (*n* = 3).						
	MOLT-3	HepG-2	A549	HuCCA-1	HeLa	MDA-MB-231	MRC-5
1	17.86 ± 0.26	12.71 ± 1.67	20.04 ± 1.52	21.32 ± 1.84	ND	ND	40.13 ± 1.23
2	32.36 ± 1.08	44.78 ± 3.79	38.59 ± 0.24	37.92 ± 3.01	ND	ND	60.61 ± 12.75
3	8 (27.17) ^a^	44.61 ± 9.76	9 (54.35) ^a^	47.48 ± 6.96	ND	ND	24.70 (54.35) ^a^
4	0 (12.35) ^a^	20.32 (24.70) ^a^	2 (12.35) ^a^	0 (12.35) ^a^	ND	ND	5.45 (24.70) ^a^
6	20.07 ± 0.52	54.89 ± 2.04	34.73 ± 17.71	37.06 ± 0.36	ND	ND	63.36 ± 8.30
7	16.33 ± 18.02	26.46 ± 5.32	23.58 ± 2.00	25.07 ± 2.27	11.23 ± 0.05	27.03 ± 7.79	ND
8	43 (108.70) ^a^	18.23 ± 1.86	32.62 ± 3.64	103.35 ± 2.29	ND	ND	90.11 ± 6.88
10	2 (29.34) ^a^	49.75 ± 3.96	52.15 ± 3.35	44 (58.69) ^a^	ND	ND	44.77 ± 1.36
12	36.57 ± 1.11	56.50 ± 2.15	54.26 ± 3.84	66.11 ± 0.90	ND	ND	76.94 ± 10.65
13	14.90 ± 0.25	12.53 ± 0.84	17.91 ± 2.26	20.79 ± 2.32	ND	ND	37.68 ± 10.65
14	24.45 ± 3.11	41.41 ± 3.24	23.76 ± 1.87	34.41 ± 1.83	ND	ND	65.90 ± 4.54
15	12.84 ± 0.98	7.10 ± 4.76	37.93 ± 0.07	37.58 ± 1.40	6.11 ± 0.02	18.01 ± 5.74	ND
16	14.58 ± 0.36	11.31 ± 9.29	29.11 ± 8.25	34.60 ± 3.85	ND	ND	49.60 ± 6.67
Doxorubicin ^b^	ND	0.55 ± 0.12	0.92 ± 0.06	2.24 ± 0.15	0.17 ± 0.10	1.78 ± 0.54	24.85 (50.00) ^a^
Etoposide ^b^	0.07 ± 0.005	31.50 ± 15.56	ND	ND	ND	ND	ND

^a^ % inhibition (at concentration, μM); ^b^ positive control; ND: not determined.

## Supplementary Materials

The following are available online at www.mdpi.com/1660-3397/15/3/54/s1: Figure S1: ^1^H NMR spectrum of **1** in pyridine-*d*_5_, Figure S2: ^13^C NMR spectrum of **1** in pyridine-*d*_5_, Figure S3: ^1^H-^1^H COSY spectrum of **1** in pyridine-*d*_5_, Figure S4: HMBC spectrum of **1** in pyridine-*d*_5_, Figure S5: NOESY spectrum of **1** in pyridine-*d*_5_, Figure S6: HRMS spectrum of **1**, Figure S7: ^1^H NMR spectrum of **2** in CDCl_3_, Figure S8: ^13^C NMR spectrum of **2** in CDCl_3_, Figure S9: ^1^H NMR spectrum of **3** in pyridine-*d_5_*, Figure S10: ^13^C NMR spectrum of **3** in pyridine-*d_5_*, Figure S11: ^1^H NMR spectrum of **4** in CDCl_3_, Figure S12: ^13^C NMR spectrum of **4** in CDCl_3_, Figure S13: ^1^H NMR spectrum of **5** in pyridine-*d*_5_, Figure S14: ^13^C NMR spectrum of **5** in pyridine-*d*_5_, Figure S15: ^1^H NMR spectrum of **11** in CDCl_3_, Figure S16: ^13^C NMR spectrum of **11** in CDCl_3_, Figure S17: ^1^H NMR spectrum of **12** in pyridine-*d*_5_, Figure S18: ^13^C NMR spectrum of **12** in pyridine-*d*_5_, Figure S19: ^1^H NMR spectrum of **13** in CDCl_3_, Figure S20: ^13^C NMR spectrum of **13** in CDCl_3_.

## References

[1] Mahidol C., Prawat H., Sangpetsiripan S., Ruchirawat S. (2009). Bioactive scalaranes from the Thai sponge *Hyrtios gumminae*. J. Nat. Prod..

[2] Prawat H., Mahidol C., Kaweetripob W., Prachyawarakorn V., Tuntiwachwuttikul P., Ruchirawat S. (2016). Sesquiterpene isocyanides, isothiocyanates, thiocyanates, and formamides from the Thai sponge *Halichondria* sp.. Tetrahedron.

[3] Prawat H., Mahidol C., Kaweetripob W., Wittayalai W., Ruchirawat S. (2012). Iodo–sesquiterpene hydroquinone and brominated indole alkaloids from the Thai sponge *Smenospongia* sp.. Tetrahedron.

[4] Umeyama A., Ito S., Yoshigaki A., Arihara S. (2000). Two new 26,27-cyclosterols from the marine sponge *Strongylophora corticata*. J. Nat. Prod..

[5] Sun H.H., Cross S.S., Gunasekera M., Koehn F.E. (1991). Weinbersterol disulfates A and B, antiviral steroid sulfates from the sponge *Petrosia weinbergi*. Tetrahedron.

[6] Mattia C.A., Mazzarella L., Puliti R., Sica D., Zollo F. (1978). X-ray crystal structure determination of petrosterol p-bromobenzoate. A revision. Tetrahedron Lett..

[7] Gabriel A.F., Li Z., Kusuda R., Tanaka C., Miyamoto T. (2015). Six new polyacetylenic alcohols from the marine sponges *Petrosia* sp. and *Halichondria* sp.. Chem. Pharm. Bull..

[8] Watanabe K., Tsuda Y., Hamada M., Omori M., Mori G., Iguchi K., Naoki H., Fujita T., Van Soest R.W.M. (2005). Acetylenic strongylodiols from a *Petrosia* (*Strongylophora*) Okinawan marine sponge. J. Nat. Prod..

[9] Kim J.S., Lim Y.J., Im K.S., Jung J.H., Shim C.J., Lee C.O., Hong J., Lee H. (1999). Cytotoxic polyacetylenes from the marine sponge *Petrosia* sp.. J. Nat. Prod..

[10] Hitora Y., Takada K., Okada S., Ise Y., Matsunaga S. (2011). (−)-Duryne and its homologues, cytotoxic acetylenes from a marine Sponge *Petrosia* sp.. J. Nat. Prod..

[11] Horikawa K., Yagyu T., Yoshioka Y., Fujiwara T., Kanamoto A., Okamoto T., Ojika M. (2013). Petrosiols A–E, neurotrophic diyne tetraols isolated from the Okinawan sponge *Petrosia strongylata*. Tetrahedron.

[12] Hitora Y., Takada K., Okada S., Matsunaga S. (2011). Miyakosynes A–F, cytotoxic methyl branched acetylenes from a marine sponge *Petrosia* sp.. Tetrahedron.

[13] Lee J.-S., Abdjul D.B., Yamazaki H., Takahashi O., Kirikoshi R., Ukai K., Namikoshi M. (2015). Strongylophorines, new protein tyrosine phosphatase 1B inhibitors, from the marine sponge *Strongylophora strongilata* collected at Iriomote Island. Bioorg. Med. Chem. Lett..

[14] Hoshino A., Mitome H., Miyaoka H., Shintani A., Yamada Y., Van Soest R.W.M. (2003). New strongylophorines from the Okinawan marine sponge *Petrosia* (*Strongylophora*) *corticata*. J. Nat. Prod..

[15] Noda A., Sakai E., Kato H., Losung F., Mangindaan R.E.P., de Voogd N.J., Yokosawa H., Tsukamoto S. (2015). Strongylophorines, meroditerpenoids from the marine sponge *Petrosia corticata*, function as proteasome inhibitors. Bioorg. Med. Chem. Lett..

[16] Ramesh P., Srinivasa Reddy N., Venkateswarlu Y. (1999). A new 1,2-dihydroisoquinoline from the sponge *Petrosia similis*. J. Nat. Prod..

[17] Tsukamoto S., Takeuchi T., Kawabata T., Kato H., Yamakuma M., Matsuo K., El-Desoky A.H., Losung F., Mangindaan R.E.P., de Voogd N.J. (2014). Halenaquinone inhibits RANKL-induced osteoclastogenesis. Bioorg. Med. Chem. Lett..

[18] Nukoolkarn V.S., Saen-oon S., Rungrotmongkol T., Hannongbua S., Ingkaninan K., Suwanborirux K. (2008). Petrosamine, a potent anticholinesterase pyridoacridine alkaloid from a Thai marine sponge *Petrosia n*. sp.. Bioorg. Med. Chem..

[19] Kobayashi M., Chen Y.-J., Higuchi K., Aoki S., Kitagawa I. (1996). Marine natural products. XXXVII. Aragusterolketals A and C, two novel cytotoxic steroids from a marine sponge of *Xestospongia* sp.. Chem. Pharm. Bull..

[20] Iguchi K., Fujita M., Nagaoka H., Mitome H., Yamada Y. (1993). Aragusterol A: A potent antitumor marine steroid from the Okinawan sponge of the genus, *Xestospongia*. Tetrahedron Lett..

[21] Kobayashi J., Ishida K., Naitoh K., Shigemori H. (1993). Xestokerols A, B, and C, new C_29_ steroids with a cyclopropane ring from the Okinawan marine sponge *Xestospongia* sp.. J. Nat. Prod..

[22] Nguyen X.C., Longeon A., Pham V.C., Urvois F., Bressy C., Trinh T.T.V., Nguyen H.N., Phan V.K., Chau V.M., Briand J.-F. (1993). Antifouling 26,27-cyclosterols from the Vietnamese marine sponge *Xestospongia testudinaria*. J. Nat. Prod..

[23] Tung N.H., Minh C.V., Kiem P.V., Huong H.T., Ha T.T., Dat N.T., Nhiem N.X., Cuong N.X., Hyun J.-H., Kang H.-K. (2009). A new C_29_-sterol with a cyclopropane ring at C-25 and 26 from the Vietnamese marine sponge *Lanthella* sp.. Arch. Pharm. Res..

[24] Zhang H., Timmermann B.N. (2016). Withanolide structural revisions by ^13^C NMR spectroscopic analysis inclusive of the γ-gauche effect. J. Nat. Prod..

[25] Mandeau A., Debitus C., Ariès M.F., David B. (2005). Isolation and absolute configuration of new bioactive marine steroids from *Euryspongia n.* sp.. Steroids.

[26] Doyle A., Griffiths J.B. (1997). Mammalian Cell Culture: Essential Techniques.

[27] Carmichael J., DeGraff W.G., Gazdar A.F., Minna J.D., Mitchell J.B. (1987). Evaluation of a tetrazolium-based semiautomated colorimetric assay: Assessment of chemosensitivity testing. Cancer Res..

