# Supplementary material for: Sterols from Thai Marine Sponge Petrosia (Strongylophora) sp. and Their Cytotoxicity
Source: Mar Drugs. 2017 Feb 23;15(3):54. doi: 10.3390/md15030054 (PMC5367011; doi:10.3390/md15030054)
Supplement: Supplementary file 1 [file marinedrugs-15-00054-s001.pdf]

Supplementary Materials: Sterols from Thai Marine Sponge *Petrosia* (*Strongylophora*) sp. and Their Cytotoxicity

Phanruethai Pailee, Chulabhorn Mahidol, Somsak Ruchirawat and Vilailak Prachyawarakorn

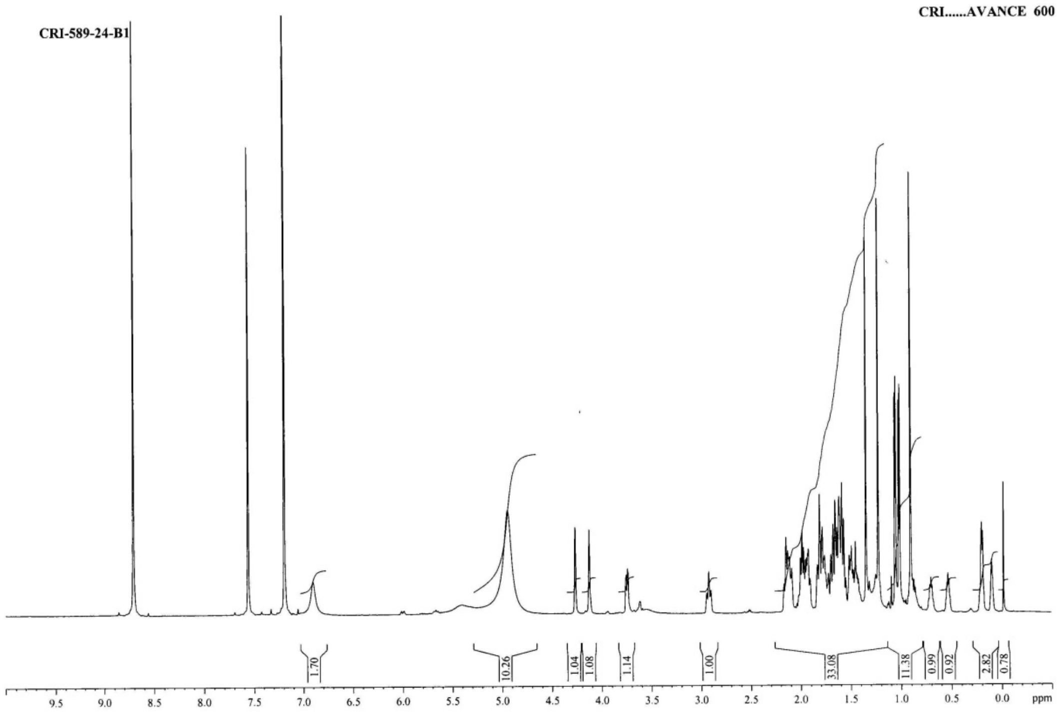

Figure S1. ^{1}H NMR spectrum of **1** in pyridine-*d5*

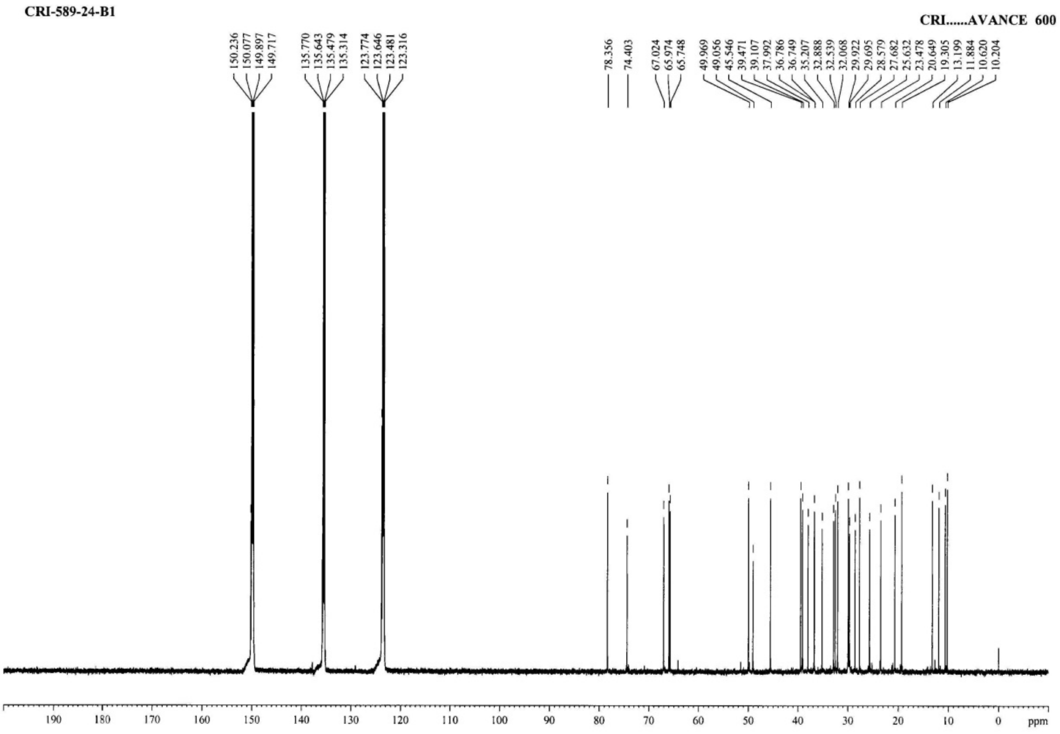

Figure S2. ^{13}C NMR spectrum of **1** in pyridine-*d5*

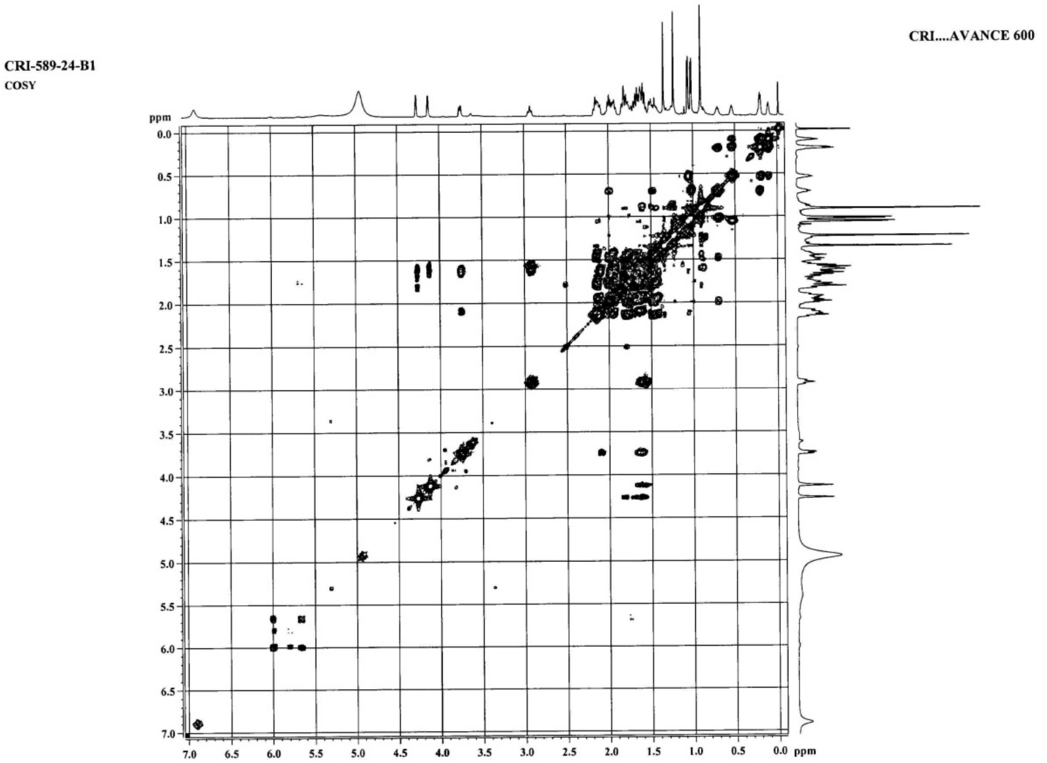

Figure S3. ^{1}H-^{1}H COSY spectrum of **1** in pyridine-*d5*

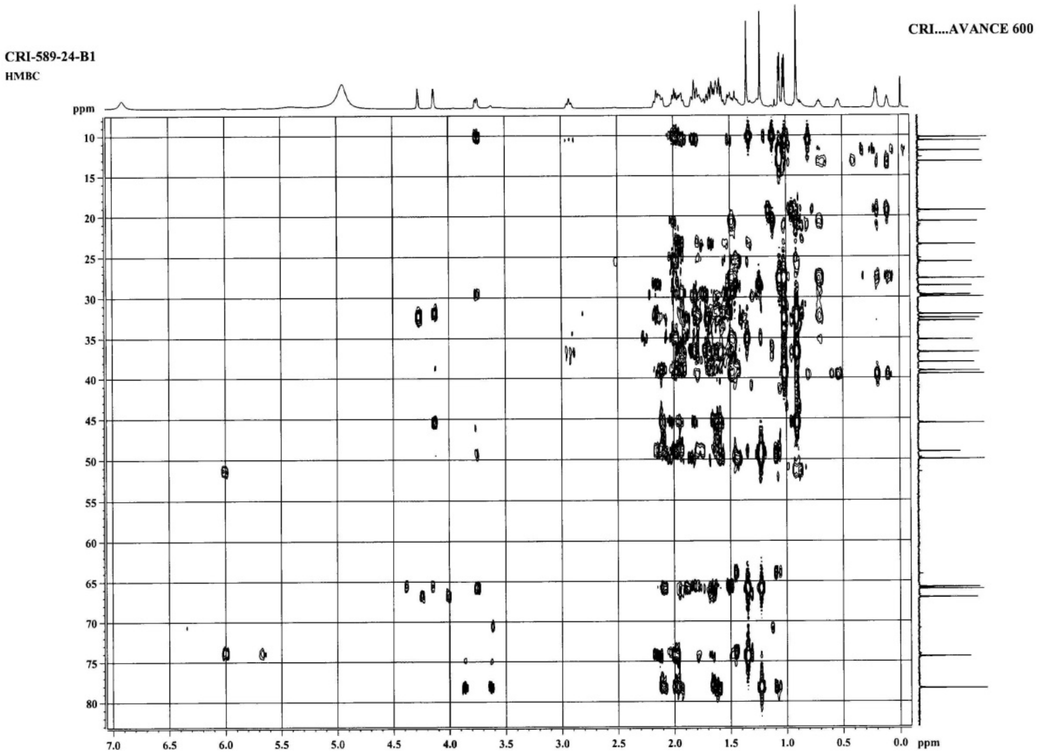

Figure S4. HMBC spectrum of **1** in pyridine-*d5*

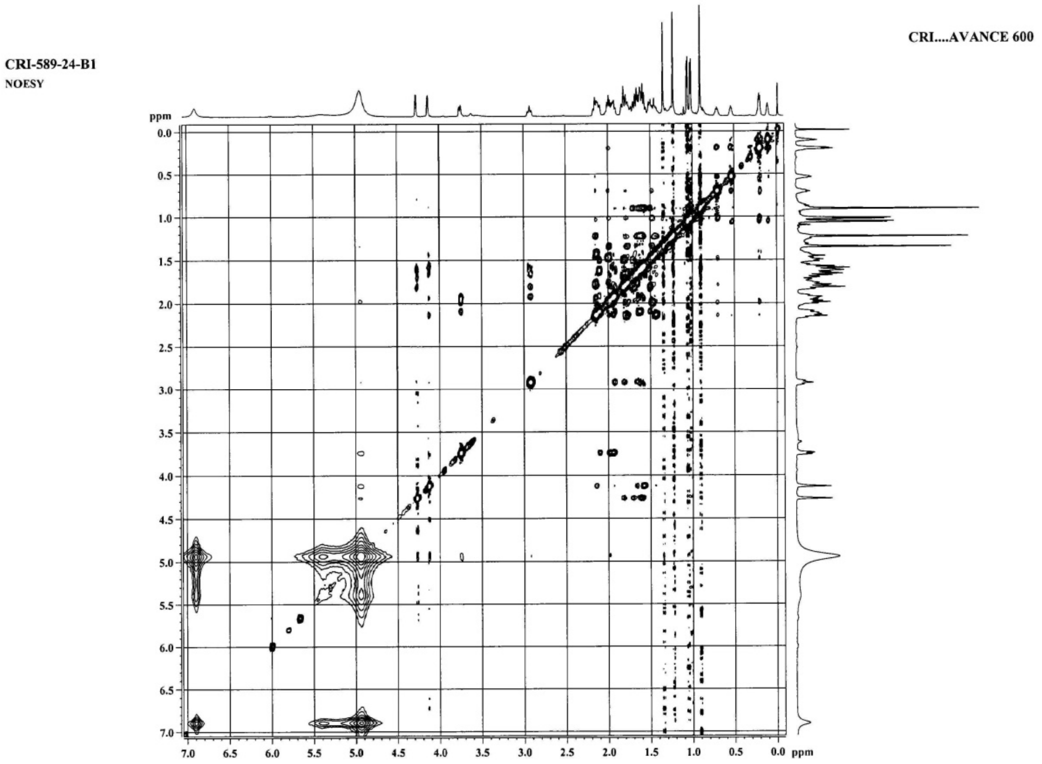

Figure S5. NOESY spectrum of **1** in pyridine-*d5*

Mass Spectrum List Report

Analysis Info

Analysis Name	TOFCRI013162 Vilailuk CRI-589-24-B1 A-.d	Acquisition Date	6/5/2012 11:36:06 AM
Method	apci_neg_low-nitirat1.m	Operator	Administrator
Sample Name	APCIneg Operator:Nitirat	Instrument	micrOTOF 74

Acquisition Parameter

Source Type	APCI	Ion Polarity	Negative	Set Corrector Fill	56 V
Scan Range	n/a	Capillary Exit	-90.0 V	Set Pulsar Pull	409 V
Scan Begin	120 m/z	Hexapole RF	120.0 V	Set Pulsar Push	409 V
Scan End	1000 m/z	Skimmer 1	-30.0 V	Set Reflector	1300 V
		Hexapole 1	-24.0 V	Set Flight Tube	9000 V
				Set Detector TOF	2250 V

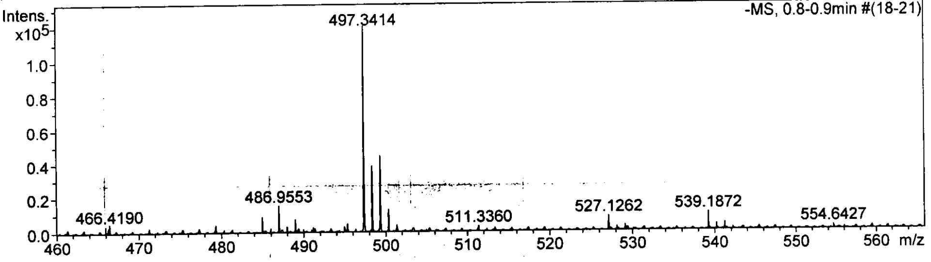

#	m/z	Res.	S/N	I	FWHM
1	145.3359	2363	742.2	51189	0.0615
2	145.5963	2178	297.0	20500	0.0669
3	145.8546	2139	286.4	19767	0.0682
4	146.1159	2447	115.2	7963	0.0597
5	197.0226	6642	108.3	13060	0.0297
6	213.0172	6834	141.4	19642	0.0312
7	215.0323	6999	1175.8	164071	0.0307
8	216.0359	6863	84.4	11748	0.0315
9	217.0303	7154	393.3	54172	0.0303
10	229.0484	7002	1606.8	202711	0.0327
11	230.0520	7226	135.5	17014	0.0318
12	231.0462	7235	545.5	67812	0.0319
13	441.0079	9134	493.3	75196	0.0483
14	442.0107	8973	102.3	15871	0.0493
15	443.0050	8899	452.4	70480	0.0498
16	444.0082	9113	97.3	15434	0.0487
17	445.0026	9062	155.1	24775	0.0491
18	484.9590	8722	49.4	8960	0.0556
19	486.9553	9333	86.2	15433	0.0522
20	497.3414	9155	696.9	120589	0.0543
21	498.3441	9436	224.7	38896	0.0528
22	499.3398	9194	258.9	44686	0.0543
23	500.3438	8871	73.9	12845	0.0564
24	527.1262	9300	51.8	8341	0.0567
25	539.1872	9120	70.9	10365	0.0591
26	655.9903	9563	102.9	8907	0.0686
27	718.8331	42208	111.9	8778	0.0170
28	955.9719	10931	713.8	37827	0.0875
29	957.9704	10694	236.2	12604	0.0896
30	1025.6957	50067	223.3	9170	0.0205

Figure S6. HRMS spectrum of **1**

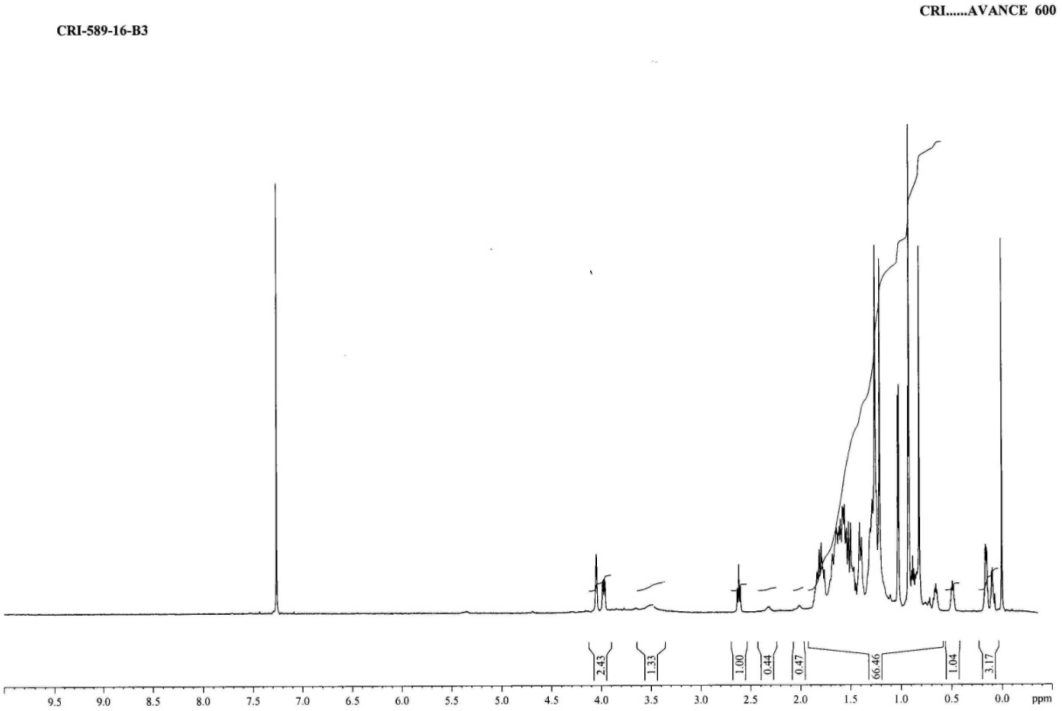

Figure S7. ^{1}H NMR spectrum of **2** in $CDCl_3$

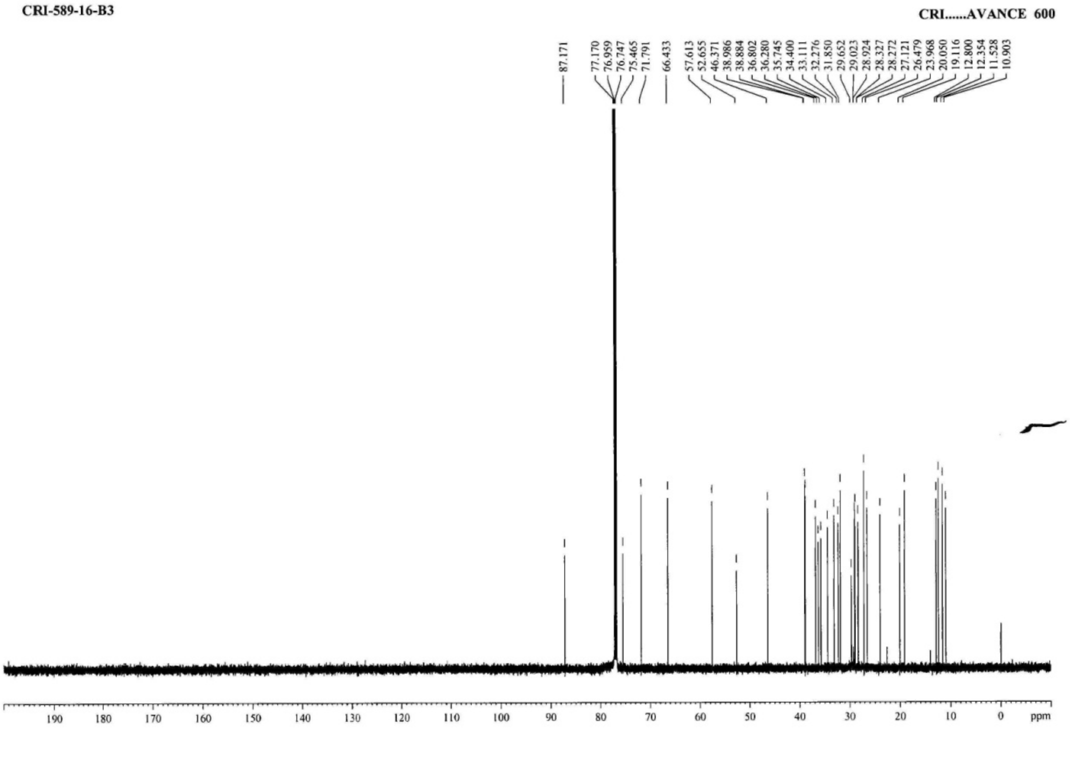

Figure S8. ^{13}C NMR spectrum of **2** in $CDCl_3$

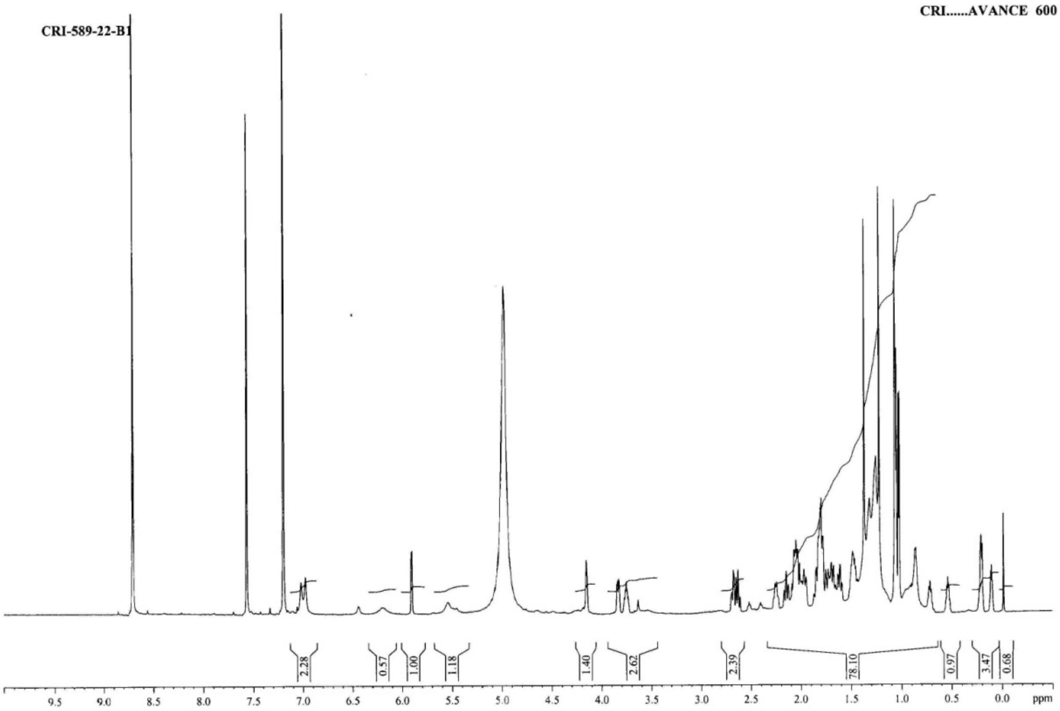

Figure S9. ^{1}H NMR spectrum of **3** in pyridine-*d5*

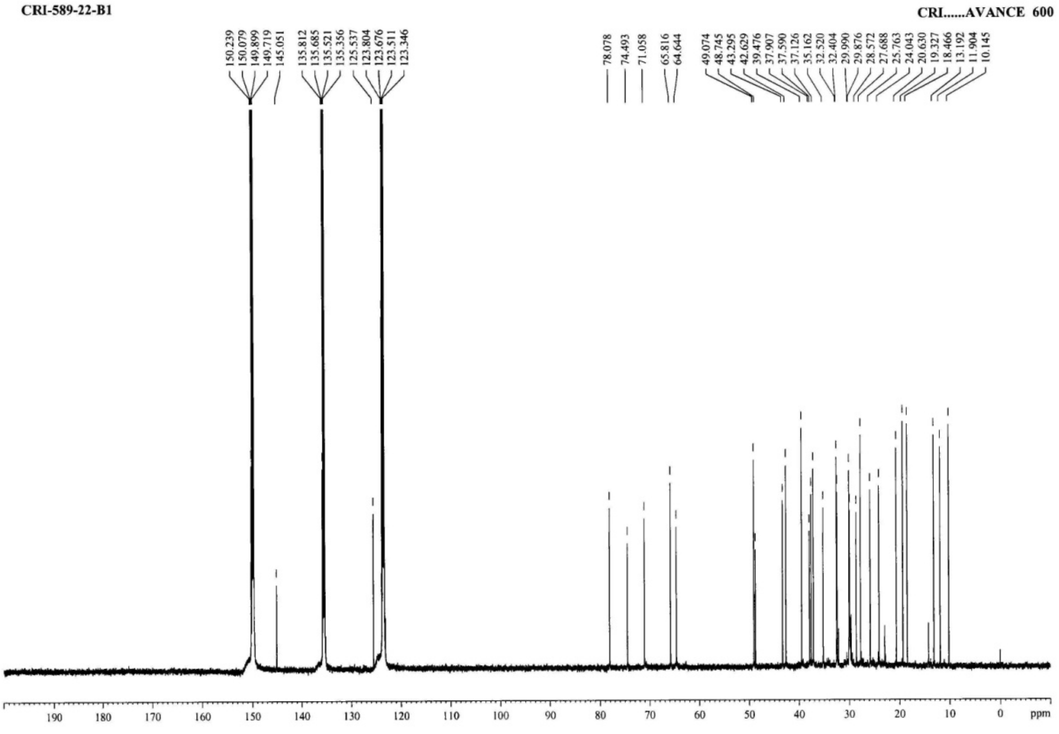

Figure S10. ^{13}C NMR spectrum of **3** in pyridine-*d5*

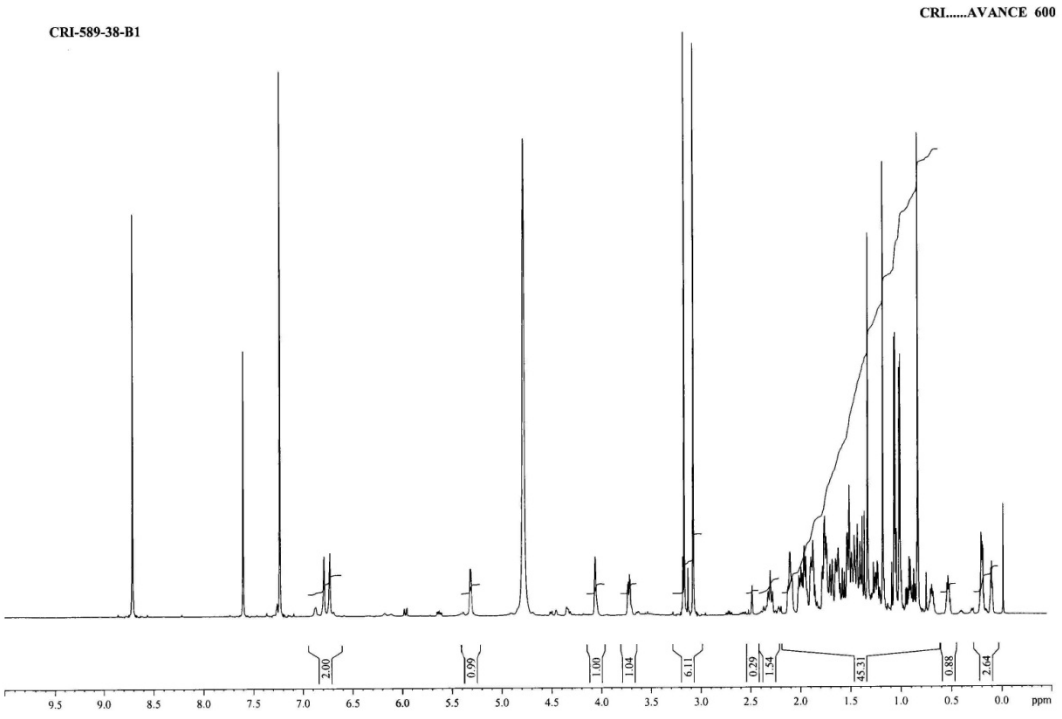

Figure S11. ^{1}H NMR spectrum of **4** in $CDCl_3$

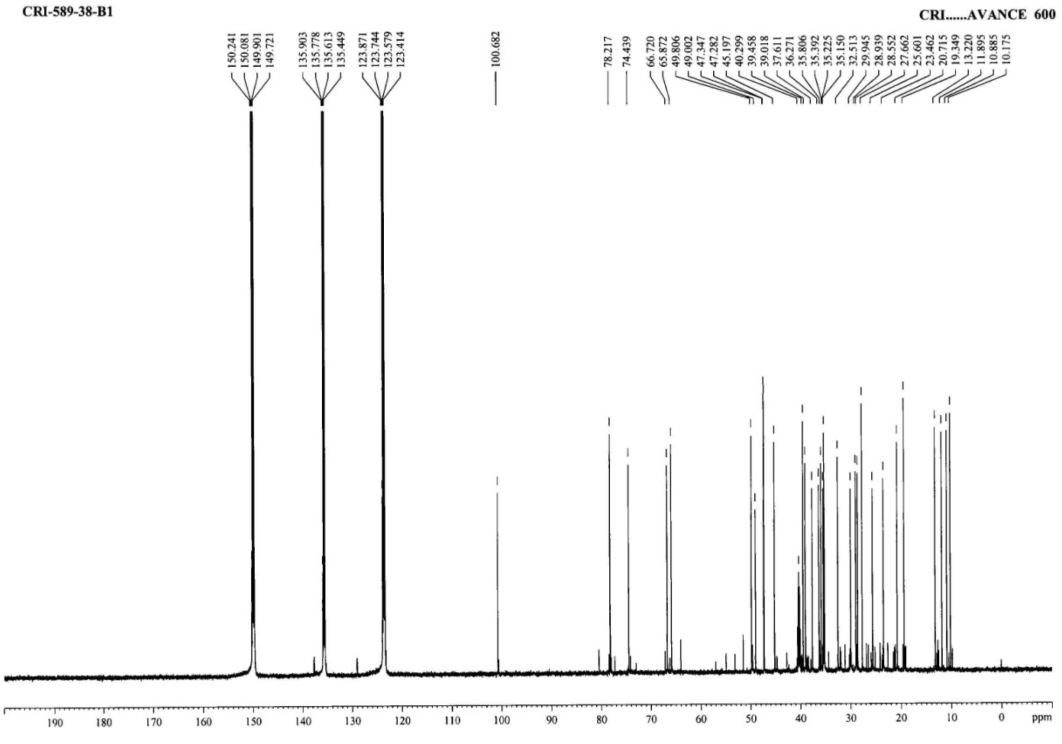

Figure S12. ^{13}C NMR spectrum of **4** in $CDCl_3$

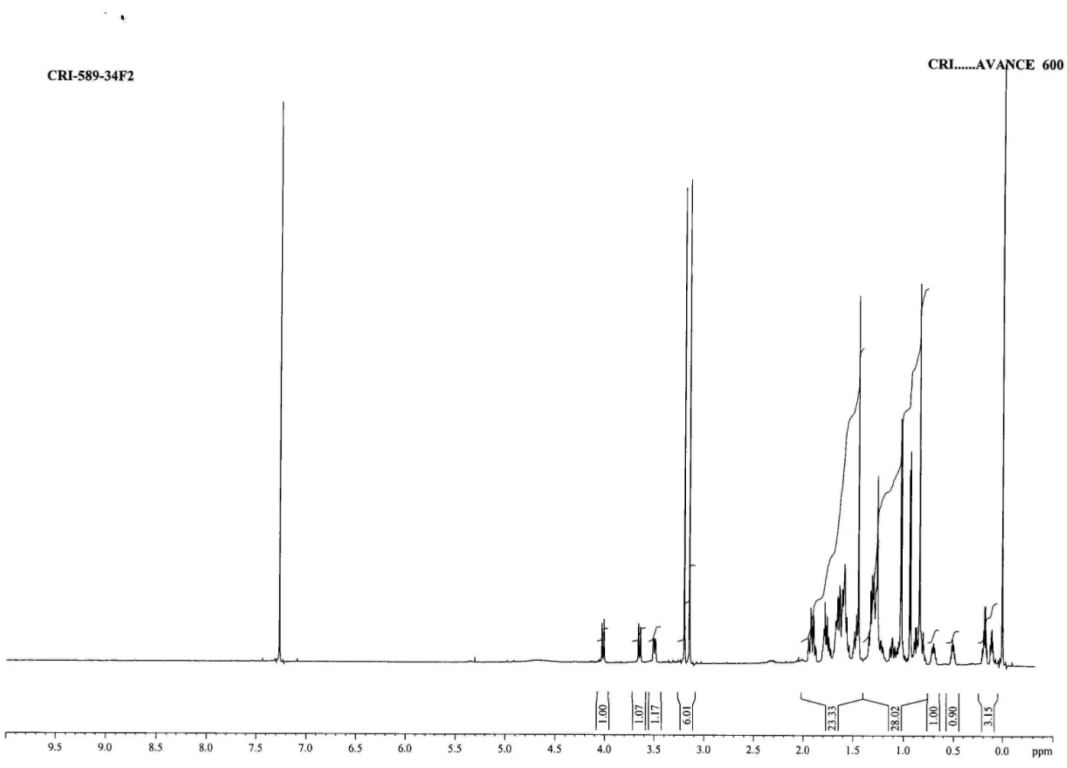

Figure S13. ^{1}H NMR spectrum of **5** in pyridine-*d5*

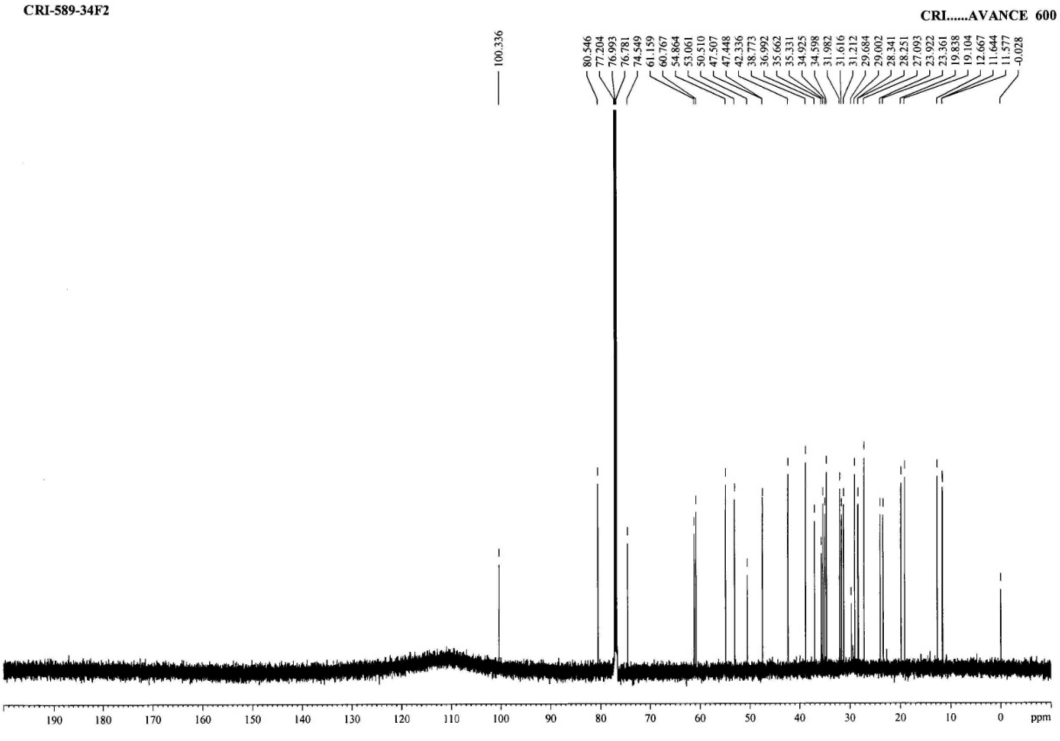

Figure S14. ^{13}C NMR spectrum of **5** in pyridine-*d5*

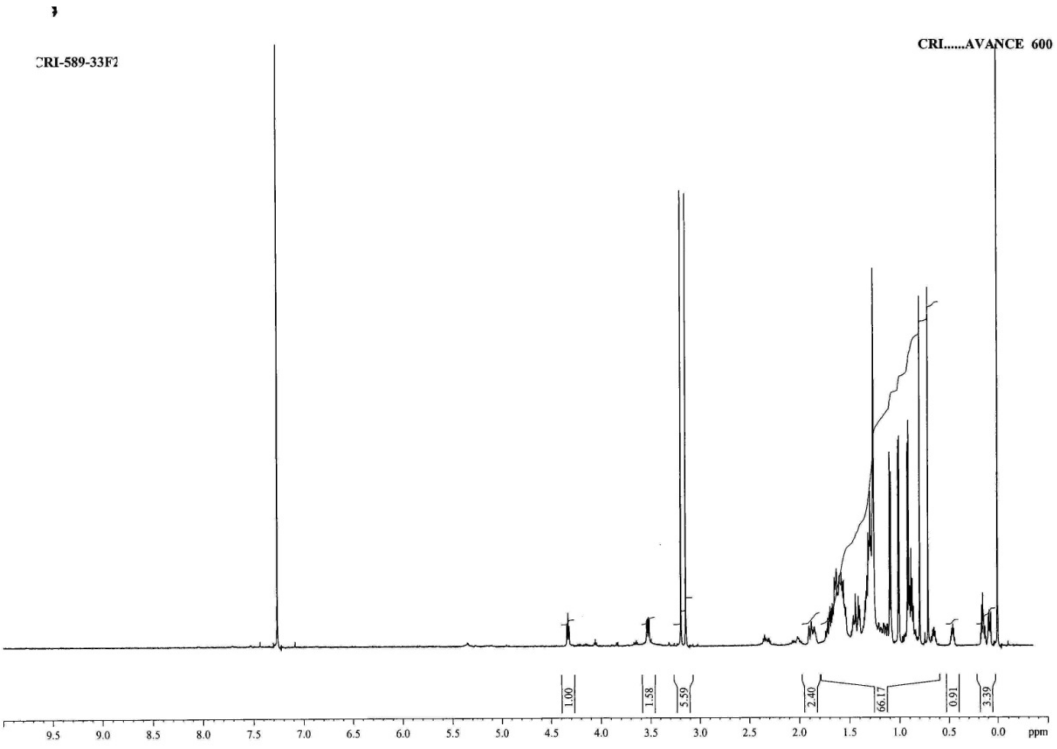

Figure S15. ^{1}H NMR spectrum of **11** in $CDCl_3$

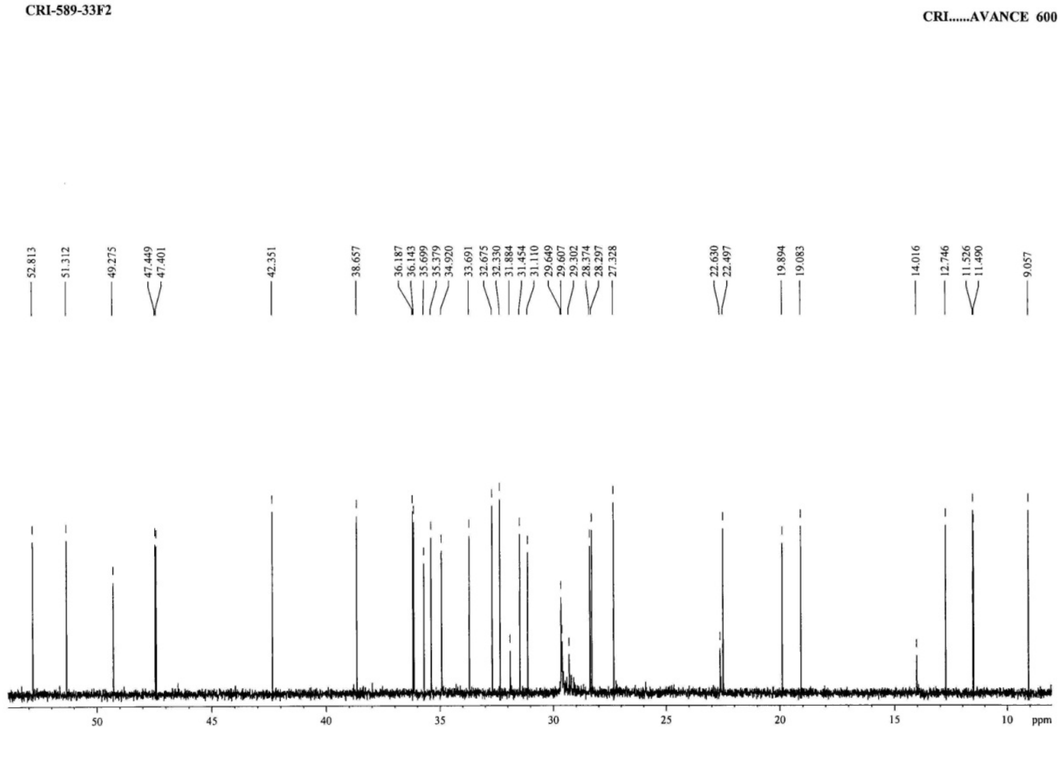

Figure S16. ^{13}C NMR spectrum of **11** in $CDCl_3$

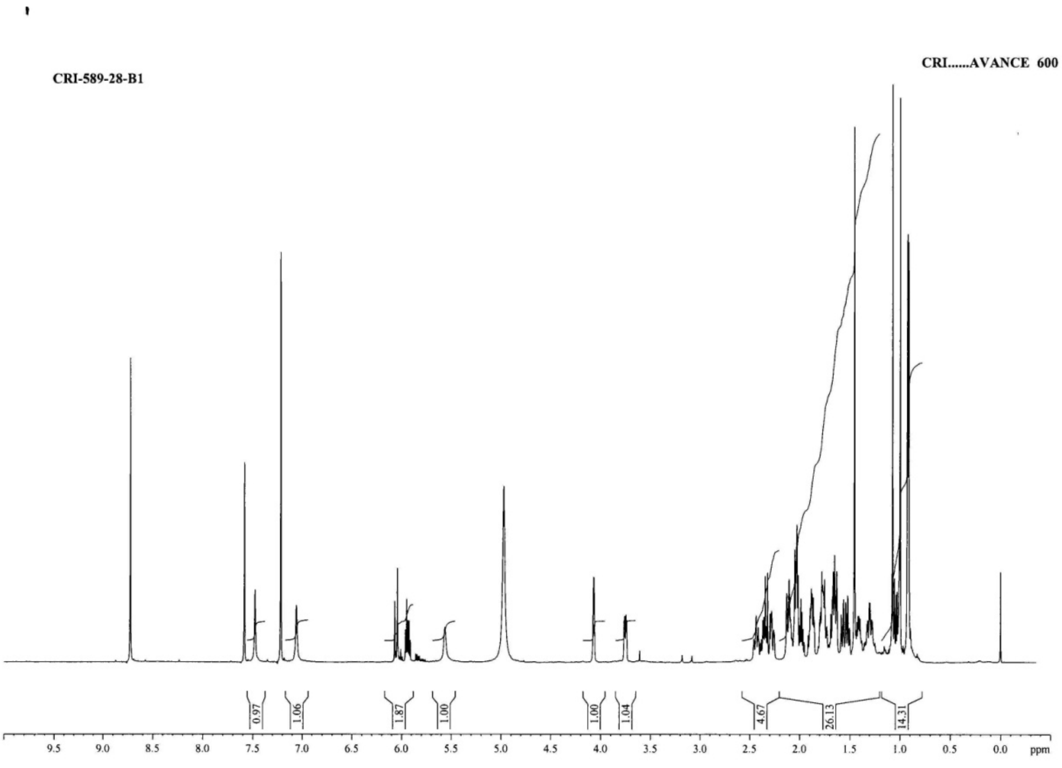

Figure S17. ^{1}H NMR spectrum of **12** in pyridine-*d5*

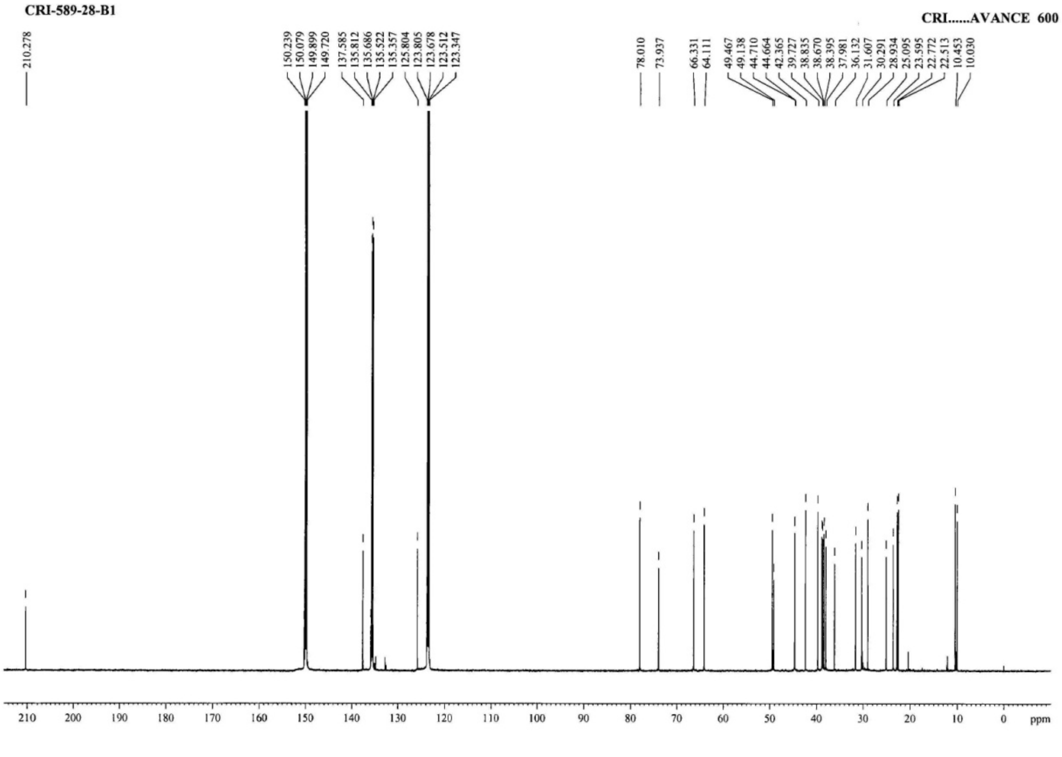

Figure S18. ^{13}C NMR spectrum of **12** in pyridine-*d5*

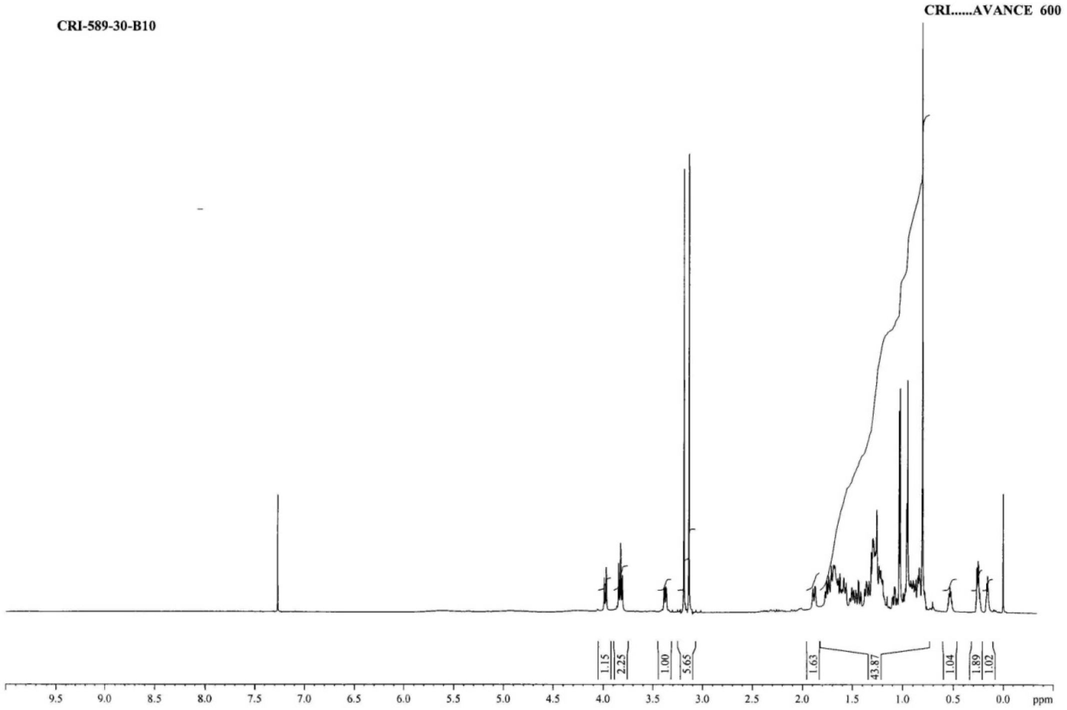

Figure S19. ^{1}H NMR spectrum of **13** in $CDCl_3$

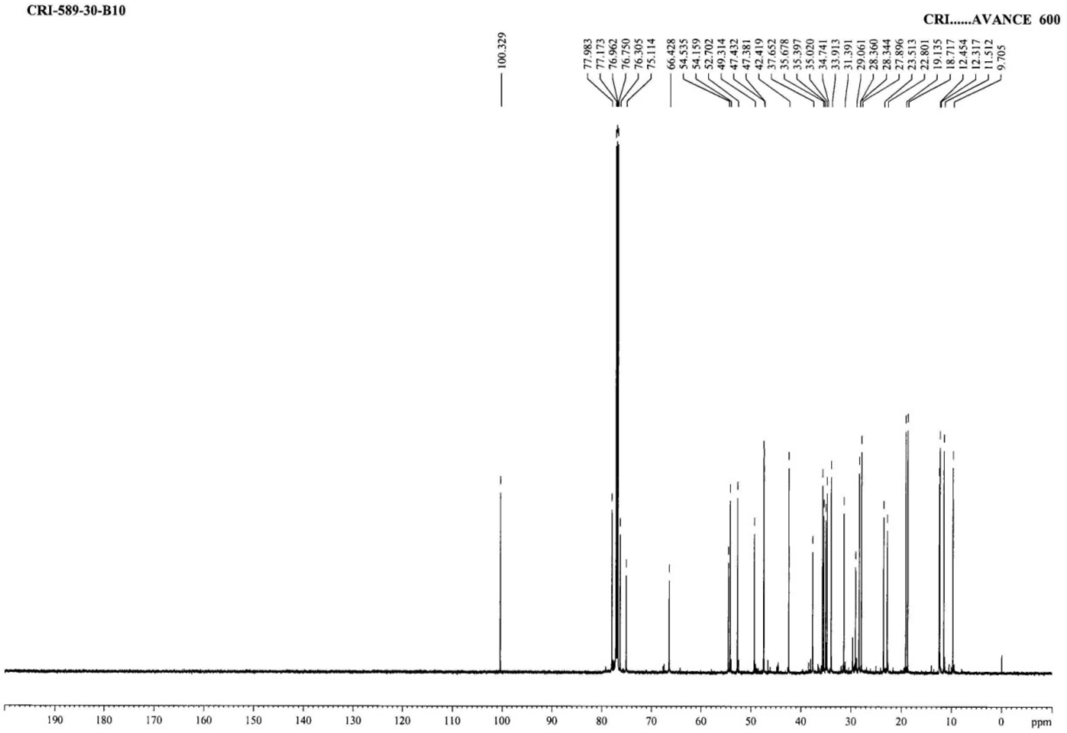

Figure S20. ^{13}C NMR spectrum of **13** in $CDCl_3$
